# Editorial for the Special Issue on Femtosecond Laser Micromachining for Photonics Applications

**DOI:** 10.3390/mi11110994

**Published:** 2020-11-06

**Authors:** Andrea Crespi, Giacomo Corrielli

**Affiliations:** Dipartimento di Fisica–Politecnico di Milano, and Istituto di Fotonica e Nanotecnologie–Consiglio Nazionale delle Ricerche (IFN-CNR), Piazza Leonardo da Vinci, 32, 20133 Milano, Italy

Femtosecond laser pulses have proven, in the recent years, their formidable potential as a micromachining tool applicable to a variety of materials. In particular, femtosecond laser processing has shown unique capabilities in altering the optical properties of the bulk of transparent dielectric substrates in a permanent fashion and with a micrometric resolution, thus allowing the manufacture of innovative integrated devices for photonics applications.

One important application of this technique is the direct and rapid inscription of integrated waveguide circuits with three-dimensional layouts. In the last decade, such circuits have found impressive applications in diverse fields, which include optical sensing, telecommunications, astrophotonics, and quantum photonics. Femtosecond laser pulses have also been exploited to locally engineer the birefringence of the substrate and to produce microstructured birefringent plates.

Optical structures inscribed in the bulk may also be combined with three-dimensional microstructuring of the substrate, exploiting laser ablation or laser-assisted chemical etching. In addition, multiphoton polymerization of photoresists, obtained by means of ultrashort laser pulses, enables additive manufacturing of microstructures with feature size below the diffraction limit, opening up novel perspectives in nanophotonics. Furthermore, femtosecond laser pulses can be used to pattern hard chromium masks, employed with planar technologies to fabricate integrated optical circuits in novel material configurations. 

The community researching in this field is growing at a steady pace: optical devices produced by this technology are amazingly increasing in complexity, and novel ideas are continually being presented. Accordingly, this Special Issue contains eight research articles on technological advances and novel applicative concepts of femtosecond laser micromachining, with a focus on optics and photonics.

Two papers, by Romero et al. [1] and Skryabin et al. [2], demonstrate waveguide devices directly inscribed in crystalline substrates. In such materials, laser irradiation produces a negative refractive-index modification and waveguides are realized using a depressed-cladding approach. In particular, in Ref. [1], a circular waveguide taper is reported in Nd:YAG crystal, while Ref. [2] shows directional couplers with three-dimensional geometries realized in Tm^3+^:YAG.

Complex micro-optic devices, based on femtosecond-laser assisted chemical etching, are presented in papers by Ross et al. [3] and by Nazir and Bellouard [4]. Ross et al. fabricate a miniaturized probe for optical biopsy, which includes microlenses and hollow slots for optical fibers, ensuring relative alignment. This device allows for efficient signal delivery and collection during a Raman spectroscopy-based optical biopsy. Nazir and Bellouard realize a micromirror suspended on gimbal flexure mounts: the entire micro-mechanical structure is monolithic and can be tuned by using the femtosecond laser itself. In fact, laser irradiation of specific points of the kinematic parts, after fabrication, cause local and permanent deformations, enabling precise adjustment of the mirror orientation.

Lin et al. [5] use femtosecond laser pulses to pattern chromium masks, deposited on a lithium-niobate-on-insulator substrate. Chemomechanical polishing is employed to transfer the pattern on the substrate and produce lithium-niobate ridge waveguides. In their article, Lin et al. are able to characterize with high precision the propagation loss of the resulting waveguides, using a novel method.

The birefringence properties of laser-induced modifications in fused silica are studied by Tian et al. [6]. The authors focus their study on the comparison between birefringence due to nanogratings with stress-induced birefringence. In addition, they exploit their results for the fabrication of half waveplates with femtosecond laser micromachining, with particular care paid to the UV-visible wavelength range.

Finally, two papers regard additive manufacturing. Yan et al. [7] demonstrate the high-speed nanofabrication of a 3D Fresnel microlens array based on two-photon polymerization. The use of a spatial light modulator allows the authors to perform careful focal field engineering, for fabricating each lens by a single sample scan, thus paving the way towards extending the fabrication technique to mass production. On the other hand, He et al. [8] combine two-photon polymerization with the two-beams stimulated emission depletion technique for increasing the spatial resolution of the nanofabrication technique. Using this approach, the authors are able to demonstrate the polymerization of single lines in PETA photoresist with the remarkable width of 45 nm. 

We are grateful to all authors who submitted their work to this Special Issue; we would also like to acknowledge the reviewers for dedicating their time and for helping to improve the quality of the published manuscripts.
